# Research on sports reform in the network era based on educational psychology in the context of the COVID-19

**DOI:** 10.3389/fpsyg.2023.1106483

**Published:** 2023-02-03

**Authors:** QI Fan

**Affiliations:** ^1^Faculty of Education, Shaanxi Normal University, Xi’an, China; ^2^College of Chemistry and Chemical Engineering, Xi’an Shiyou University, Xi’an, China

**Keywords:** Internet+, college sports, online education, reform, educational psychology

## Abstract

**Introduction:**

In the context of the continuous progress of the “Internet+” era and the exceptionally severe outbreak of the Covid-19, online education confronts many challenges and also brings unlimited development opportunities.

**Methods:**

In this paper, taking six universities with sports majors in Chongqing as the research objects, we analyze the current situation and problems of the development and construction of online education resources for sports in colleges and universities by browsing the official websites of colleges and universities, investigating the cooperation between colleges and universities and Massive Open Online Course (MOOC), and conducting a questionnaire survey on students.

**Results:**

The results show that it is necessary to innovate the traditional inefficient classroom teaching model based on the values of adapting to the characteristics of the “Internet+” era and learning advanced teaching rules, thus optimizing teaching effectiveness and fostering students’ independent learning, thinking and innovation.

**Discussion:**

We investigated students’ online learning habits, their experience of using online education platform and the difficulties they encountered in the process, based on which two issues were raised: learning needs and learning experience.

## 1. Introduction

It is well known that the Internet is a double-edged sword, and how to use it to build on its strengths and avoid its weaknesses to better serve college sports is a problem that every educator should be concerned about (Wegerif, 2019). The application and popularity of the Internet has promoted the construction and development of a number of online teaching platform websites, among which the more famous ones are Massive Open Online Course (MOOC), Hujiang Online School, and Pandora English. Some of these platforms are websites established for individual disciplines, while others are integrated disciplinary education platforms. MOOC is a large integrated Internet learning platform that offers multi-disciplinary courses, and is followed by many double first-rate universities and well-known scholars in China, aiming to meet the needs of learning users in various disciplines (De Notaris et al., 2021). However, there are still some characteristics of outmoded teaching concepts and contents in physical education teaching in colleges and universities in some countries.

Before the “Covid-19” epidemic hit, many disciplines in colleges and universities had not yet applied online teaching methods to teaching, and the development of online teaching was slow. Although some subjects are not suitable for online teaching, no attempt has been made, resulting in a stagnation in the development of online teaching. At the beginning of 2020, the “Covid-19” epidemic hit the traditional offline teaching, which brought opportunities and challenges to China’s education model. With the continuous development of Internet technology, online teaching will be one of the factors promoting educational reform. In this “new era” of education caused by the intersection of “information technology” and “Covid-19,” new changes have taken place in the form of teaching. During the epidemic, comprehensive online teaching was carried out to promote the development of education reform in the new era, and bring new opportunities for the development of subjects that were originally practice-based and not suitable for online teaching. It is helpful to clarify the positioning and development trend of future teaching, promote the design and implementation of teaching links, and develop high-quality teaching.

Physical education is a secondary subject of education. Students majoring in physical education and physical education normal students in colleges and universities are typical teaching executive groups in this discipline. However, the single limitation of teachers’ teaching methods, traditional and outdated learning forms, and students’ passive absorption of knowledge and information are still problems that need to be solved urgently in the training process. Under the influence of the “Internet +” era and the epidemic, the reform of physical education is an effective way to accelerate the modernization of education. Utilize modern Internet information technology, adopt methods such as building and upgrading online education platforms, and optimizing online college physical education teaching resources to innovate and improve education and teaching methods, improve students’ learning efficiency, and reduce the cost of knowledge and information acquisition. This is an important research focus of building a high-level physical education teacher team.

At present, Internet technology organizations, technical teams, and university groups in various countries and regions are paying attention to the construction of online teaching resources sharing platform, and have made some research based on it again. Many different types of universities and colleges have also created exclusive online teaching and learning resource sharing platforms. However, Internet technology is a relatively new field that will be more useful and valuable when combined with the education system and subject knowledge. In addition, such platforms are not well developed in terms of theoretical and practical issues, narrowing the educational field to the sports category, with even fewer relevant and mature research results (Sosa-Díaz and Fernández-Sánchez, 2020).

In the training process of college students majoring in physical education or normal students majoring in physical education, China traditionally focuses more on the totality of the discipline and adopts a batch and group approach to education, paying less attention to individual differences. At the same time, more attention and resources are put on practical training, while relatively little attention is paid to the construction of physical education knowledge, social sports spiritual civilization, and campus sports culture atmosphere. No form of sports can adapt to the characteristics of different regions, different industries, and different people, and cannot better meet the subjective needs of students, making them more passive in absorbing knowledge. In addition, imperfect policies and regulations for the construction of network platforms, uneven quality of online teaching resources, lack of unified standards and specifications for shared courses, and insufficient attention to users’ needs and opinions are also problems that need to be solved. Therefore, constructing and optimizing the online physical education teaching resources sharing platform in colleges and universities is a topic worthy of in-depth study (Hsu, 2021).

In addition, physical education in colleges and universities in the new era is facing great opportunities and challenges (Lemes et al., 2022). In modern higher education, where new technologies are emerging, physical education should also keep up with the times and new approaches and steps need to be taken. In the Internet+ era, physical education teachers should not only store rich expertise in physical education, but also keep pace with teachers of other disciplines in terms of mastering and applying new technologies. In modern university teaching, the use of Internet+ technology has penetrated into the work and study of every teacher and student. Therefore, in terms of the integration of sports and Internet+ in this emerging but not unfamiliar field, the construction of an online educational resource base has naturally become a top priority in the process of building software infrastructure in universities (Li, 2021). The development and also the online educational resource library of the school is gradually related to the quality and level of school operation. As a part of the educational resource base of colleges and universities, the development and construction of online sports resources is imperative. The research of this paper aims to provide a reference for improving the application effect of the construction of high-quality digital education resources for sports in China’s colleges and universities, fully reflecting the concept of paying attention to the needs of teachers and students and putting people and users first. With the continuous and deep integration of Internet and education, more network technologies have been applied and promoted in the field of education, and the construction of online sports teaching resources in colleges and universities needs to be studied more deeply.

Based on the impact of the epidemic, it provides an important opportunity for online physical education teaching. Through this study, we comprehensively analyze the situation faced by online physical education teaching during the epidemic period, conduct evaluation and reflection, and put forward development ideas for the reform of online physical education teaching. It not only makes the means for teachers and students to master knowledge more diversified, but also improves students’ awareness of physical exercise, self-learning ability, discussion ability, and increased sports participation behavior. It can also improve the application effect of online courses, and finally achieve effective teaching and achieve the purpose of students’ all-round development. This study provides a practical basis for the teaching methods adopted in emergencies in the future.

## 2. Research status

This paper explores the existing online education platforms based on the current situation of traditional education and online education, for example, the traditional campus culture has problems such as single mode of talent training, information exchange restricted by time and space, and relatively lagging culture and information circulation (Asli et al., 2020). In addition, free learning resources on existing search sites are relatively scattered and also suffer from more harmful information problems such as duplication of content, lack of information depth and content. In addition, many online education platforms also suffer from imperfect policies and regulations, lack of attention to user needs and opinions, varying levels of resources, and lack of a unified certification mechanism for quality educational resources (Yu and Jee, 2020). In terms of user perception, there is also a lack of awareness of online resource education platforms in universities, weak ability of educators to use the Internet, neglect of students’ interests, and copying traditions to achieve teaching effects. Based on this, scholars have proposed the necessity or advantages of building and optimizing the Internet teaching resource platform (hereinafter referred to as the platform). For example, in terms of campus network culture, network information spreads fast and efficiently, and online campus culture has a wide range of influences; and in terms of cross-field combination of “Internet+,” the human-centered “crowd innovation” mechanism should focus on individual needs.

Resource base construction is often considered by researchers as the most important step in building and optimizing a platform, and the resource base is also the part that reflects the core competitiveness and use value of the platform (Chen et al., 2020). First of all, there are many ways to use the content of the resource base, such as school, family, society, policy, traditional culture, and natural environment. Scholars believe that, in addition to using traditional and classical subject knowledge, more new types of resources should be discovered in combination with the use of new technologies. In addition, there is a need to maintain the pedagogical function of online educational resources by cleaning up the online environment. The content of the resource library should be aligned with the trends in sport and the teaching objectives of each university, and updated regularly to ensure that the user community can keep up with the latest information on the subject and experience a good learning process (Beard and Konukman, 2020).

The usual practice of platform safeguards is government leadership, the main role of the school, and the development of a relevant network platform system to ensure information and copyright security. In addition, the practical operation class is mainly related to the following aspects of research (Ruiz-Palmero et al., 2020): (a) platform operating environment—such as media server, web server, FTP server, and address server; (b) platform technology design—commonly used web technologies are object-oriented software engineering methods, big data technologies, Web 2.0, and cloud computing technologies. In addition, IoT technology is a technology that integrates hardware education resource information into the education resource sharing platform, and scholars have proposed methods to help optimize the online teaching platform in the related literature; and (c) platform module design—the modules in the platform are user experience areas that perform their designated functions. They are interrelated and can operate independently or be dynamically added or deleted as needed, such as system management module, information retrieval module, teacher–student interaction module, teaching evaluation module, etc. The operation and overall association of these modules are supported by web technologies in the platform design. They can better improve the management of teaching resources and integrate the process of realizing various functions for online teaching; and (d) operational structure of the platform—the principle of the platform’s retrieval structure is to rationalize multiple database modules with different objectives to their goals and resources in order to coordinate the various things that users need to retrieve. Each database module is itself an autonomous computing entity that can automatically identify key fields and targets. Finally, multiple database modules coordinate and cooperate to form a problem retrieval and resolution network. The campus network should not only be a carrier of information technology, but also a functional entity of culture, values, and education (Goad et al., 2019). Universities should not only pay attention to the technical issues of hardware and software, but also do a good job of positioning the campus network culture, improving network security, strengthening online supervision, cultivating the Internet thinking of college teachers and students, and enhancing their ability to use network technology. It is also required to deeply integrate online and offline teaching resources, increase the supply of educational resources, address the common needs of college users for quality teaching resources, and realize real-time sharing of teaching resources and online teaching experience, construct or improve the actual employment plan of students, improve the employment information database in a targeted way, and realize the organic combination of talent training and employment in enterprise units (Tabuenca et al., 2018).

From the above information, it can be found that there is a relative lack of research results combining Internet and online educational resources with college physical education. From the perspective of optimizing the quality of platform college physical education resources and improving the effectiveness of college physical education, we explore how to discover the problems and solutions of existing Internet teaching resource sharing platforms and take more specific measures, for example, comparing the advantages and disadvantages of the existing major online learning platforms in China, increasing user experience research and discussion, combining the needs of user groups, and taking appropriate methods to integrate the existing physical education resources in colleges and universities more effectively, more standardized digital resources, more convenient uploading and updating to the platform resource base, and more powerfully uniting major universities to expand the scope and influence of resources. These reflections provide space and possibilities for in-depth research in academia.

## 3. Hypotheses

Regarding the impact of online education on physical education under the above-mentioned epidemic situation, research can be carried out as follows: (1) Although research has shown that online education has been initially improved, past research has not focused on the education of college students, especially sports majors. (2) Assume that most college students are not interested in online physical education. Because the user interface is complicated, it is difficult for students to interact with teachers. (3) This paper predicts that although most college students use online education platforms with high frequency, they are not interested in online physical education.

In order to understand the current situation of online physical education resources in colleges and universities and propose optimization strategies, this paper takes Chongqing colleges and universities as an example and selects six colleges and universities including physical education teachers or physical education majors, namely Chongqing Normal University, Chongqing University of Posts and Telecommunications, Chongqing College of Arts and Science, Chongqing University of Technology and Industry, and Chongqing Second Normal College of Changjiang Normal University, to investigate the development of their online physical education teaching resources. During the epidemic period, this paper chose to browse and investigate the online homepages of each university, the cooperation with the MOOC platform, and the online questionnaire survey of physical education students in each university.

According to the survey, since none of the six universities uploaded sports-related teaching resources on the “MOOC” platform, I divided the findings of their official homepages into three parts: the timeliness of online resources, the richness of resource information, and the design and layout of web pages. In terms of the timeliness of resources, the content of most universities’ homepages lagged or even stagnated in terms of updating research information. Among them, the online journals or periodicals of three universities are in a state of frequent updating and good management and maintenance; in terms of resource information richness, all of the six universities display the information of scientific research results in the corresponding columns, but none of them provides the entrance links to the existing sports research resources or teaching resources of the school on the official homepage, e.g., teacher education resources column; in terms of webpage design and layout, all six universities adopt the traditional window format page layout. When the content displayed in the window is relatively concise, the visual effect of the page does not appear mixed. In most cases, the page layout is dense and the search bar is clear at a glance, and there is the problem that the user experience is not smooth enough and the visual effect is not good, which makes it inconvenient to search for information. Overall, the online sports resources on the official homepages of the six universities are not optimistic and cannot provide users with enough professional teaching resources for reference.

## 4. Theory support

### 4.1. Questionnaire survey and analysis

The object radiation, selection angle, and analysis method of the website homepage of six colleges and universities in Chongqing are relatively limited, and the online sports resources covered on the website do not fully represent and stand for the overall development of online sports teaching resources in colleges and universities. Therefore, the author then used the online application of Wenjuanxing to conduct a questionnaire survey on physical education students in six colleges and universities in Chongqing to investigate and explore the current situation and needs of online education of the research subjects from multiple perspectives. A total of 400 electronic questionnaires were collected, and 63 invalid questionnaires with too short response time and consistent answers were excluded. According to the third question, 32 invalid samples did not contact the online education platform, totaling 95 invalid samples, and 305 valid samples. The survey subjects were all freshmen to seniors, and the number was relatively average, as shown in Table 1. The educational platforms they currently contacted and their frequency of use were investigated to understand the current number of online courses and the main purpose of using online education platforms for students, as shown in Table 2.

**Table 1:** Basic information of survey objects.

Demographic variable	Classification	Frequency	Percentage (%)
Grade	Freshman	71	23.3
	Sophomore	66	21.6
	Junior	87	28.5
	Senior	81	26.6

**Table 2:** Main results of the questionnaire.

Describe the object	Classification	Frequency	Percentage (%)
Main online education platforms	MOOC	92	30.2
	NetEase Cloud Classroom	74	24.3
	Baidu classroom	6	2
	Tencent classroom	48	15.7
	Hujiang Online School	47	15.4
	Youdao School	28	9.2
	New Oriental Online	3	1
Usage frequency	Once a day	51	16.7
	Once a week	91	29.8
	Semi-monthly	95	31.1
	Once a month	61	20
	Quarterly and above	7	2.3
Main purpose	Assisting in the study of varsity physical education courses	103	33.8
	Learning of knowledge other than physical education courses	54	17.7
	Interest in learning, hoping to increase the accumulation of knowledge in a certain field	78	25.6
	Killing time	7	2.3
	Go with the flow and learn with everyone	53	17.4
	Other	10	3.3
Number of courses studied online	1–3	131	43
	4–6	124	40.7
	7–10	46	15.1
	Greater than 10	4	1.3

### 4.2. Reliability test

The internal consistency reliability method is used to measure the correlation between each measure and the other measures in a set of measures of the same object. The measurement indicator of this method is Cronbach’s reliability coefficient. The larger the Cronbach value of the variables, the higher the correlation coefficient of the dimensions and the higher the internal consistency between each dimension. Studies have shown that when Cronbach’s alpha is between 0.65 and 0.7, it is an acceptable level; when it is between 0.7 and 0.8, it is considered to have good reliability; and when the indicator exceeds 0.8, it means that the data is ideal. For items under each dimension, items were purified by CITC values and homogeneity tests. CITC was used to measure the correlation coefficient between each measure of the same variable and other measures to evaluate each measure. If the CITC value is greater than 0.5, it indicates that the scale has a reasonable item design and good convergence, and there is no need to eliminate items. If the reliability is low, i.e., the alpha coefficient is small, the total coefficient of the corrected items can be used to purify the measurement items (Cruickshank et al., 2021).

#### 4.2.1. Reliability test of the learning needs scale

As shown in Table 3, the reliability values of Learning Environment Needs, Learning Interaction Needs, and Learning Support Needs were 0.845, 0.847, and 0.812, respectively, all of which were greater than 0.7, and the CITC values of each item in each dimension were greater than 0.5, indicating that the reliability of each dimension was reasonable, the items were consistent, and they passed the reliability test.

**Table 3:** Learning needs scale reliability checklist.

Dimensions	Items	CITC	Cronbach’s alpha, title removed	Cronbach’s alpha
Learning environment needs	E-1	0.659	0.814	0.845
	E-2	0.627	0.820	
	E-3	0.629	0.819	
	E-4	0.575	0.830	
	E-5	0.615	0.822	
	E-6	0.650	0.815	
	I-1	0.640	0.820	
Learning interaction needs	I-2	0.646	0.818	0.847
	I-3	0.676	0.810	
	I-4	0.663	0.813	
	I-5	0.650	0.817	
	S-1	0.550	0.789	
Learning support needs	S-2	0.592	0.777	0.812
	S-3	0.661	0.756	
	S-4	0.594	0.777	
	S-5	0.600	0.775	

#### 4.2.2. Reliability testing of empirical scales

As shown in Table 4, the reliability values of functional experience, emotional experience, and social experience were 0.856, 0.851, and 0.835, respectively, which were all greater than 0.7, and the CITC values of each item in each dimension were all greater than 0.5, indicating that the reliability of each dimension was reasonable, the items were consistent, and the reliability test was passed (Daum et al., 2022).

**Table 4:** Empirical scale reliability test table.

Dimensions	Items	CITC	Cronbach’s alpha, title removed alpha	Cronbach’s alpha, title removed alpha
Functionality experience	F-1	0.690	0.821	
	F-2	0.680	0.825	
	F-3	0.730	0.856	
	F-4	0.698	0.804	
			0.817	
Emotional experience	E-1	0.709	0.804	0.851
	E-2	0.725	0.788	
	E-3	0.729	0.785	
	S-1	0.651	0.816	
Social experience	S-2	0.732	0.735	0.835
	S-3	0707	0761	

## 5. Discussion

### 5.1. Validity test of the learning needs scale

First, Bartlett’s sphericity test and Kaiser-Meyer-Olkin (KMO) test were conducted using SPSS 21.0. The results showed that the Bartlett’s sphericity test^2^ value for the 16 questions in the questionnaire learning needs scale was 1750.743 (120 degrees of freedom, sig = 0.000), and there was some overlap in the information reflected by the 16 questions, which was necessary for factor analysis. The KMO test was used to check for partial correlation between the variables (Erfayliana et al., 2022). The value is between 0 and 1. The closer the KMO statistic is to 1, the stronger the partial correlation between the variables and the better the factor analysis is. As shown in Table 5, the KMO value for this part of the questionnaire is 0.829, which indicates that it is well suited for factor analysis.

**Table 5:** Table of Bartlett’s sphericity test and KMO test for demand scales.

KMO and Bartlett tests		0.829
Bartlett’s sphericity test	Cardinality last read	1750.743
	DOF	120
	Significance	0.000

Factor analysis was performed using the extraction method of principal component analysis. Six shared factors were extracted according to the method of extracting eigenvalues greater than 1. The cumulative explained variance was 58.526%, which met the criterion of explaining more than 50%. The specific calculation results are shown in Table 6.

**Table 6:** Factor analysis of the learning needs scale.

Total differences explained									
Components	Total	Initial Eigenvalue Variance %	Total %	Total	Extraction of loading squares and variances	Total %	Total	Rotational load squared and variance	Total %
1	3.977	2.4855	24.855	3.977	24.855	24.855	3.395	21.218	21.218
2	2.709	16.933	41.755	2.709	16.933	41.788	3.108	19.424	40.652
3	3.678	16.738	58.526	2.678	16.738	58.526	2.861	17.884	58.526
4	0.799	4.747	63.273						
5	0.715	4.481	67.745						
6	0.648	4.053	71.798						
7	0.622	3.889	75.686						
8	0.857	3.672	79.358						
9	0.533	3.332	82.690						
10	0.498	3.115	85.805						
11	0.454	2.837	88.642						
12	0.403	2.516	91.158						
13	0.382	2.388	93.546						
14	0.370	2.312	95.859						
15	0.336	2.097	97.956						

Extraction method: Principal component analysis.

To further clarify the structure of each common factor, the maximum variance method was used to orthogonally rotate the indicators, and the results are shown in Table 7. The first common factor was assumed to be the learning environment needs factor, the second common factor was the learning interaction needs factor, and the third common factor was the learning support needs factor. From the factor loadings of the 16 measurement questions included in the three extracted factors, they were all above 0.5, which also exceeded the recommended critical level, reflecting the high structural validity of the questionnaire learning needs scale.

**Table 7:** Calculation results of orthogonal rotation of demand size index.

Rotating component matrix^a^			
	Component 1	Component 2	Component 3
Learning Environment Needs 1	0.777	0.041	0.042
Learning Environment Needs 2	0.752	0.038	0.032
Learning Environment Needs 3	0.747	0. 102	0.058
Learning Environment Needs 4	0.704	0.057	0.033
Learning Environment Needs 5	0.744	0.040	0.000
Learning Environment Needs 6	0.763	0.084	0.077
Learning Interaction Needs 1			
Learning Interaction Needs 2	0.051	0.779	0.028
Learning Interaction Needs 3	0.068	0.801	0.006
Learning Interaction Needs 4	0.048	0.791	0.061
Learning Interaction Needs 5	0.053	0.783	0.003
Learning Support Needs 1			
Learning Support Needs 2	0.038	0.004	0.752
Learning Support Needs 3	0.069	0.061	0.800
Learning Support Needs 4	00064	0.035	0.747
Learning Support Needs 5	0.041	0.047	0.752

Extraction method: Principal component analysis.

Rotation method: Kaiser standardized maximum variance method.

^a^Rotation converges after five iterations.

### 5.2. Empirical scale validity test

First, Bartlett’s sphericity test and KMO test were conducted using SPSS 21.0. The results showed that the χ^2^ value of Bartlett’s sphericity test for the 10 questions in the questionnaire experience scale section was 1357.370 (DOF at 40, sig = 0.000), and there was some overlap in the information reflected by the 10 questions, which was needed for factor analysis. The KMO test was used to check the partial correlation between the variables. The value is between 0 and 1. The closer the KMO statistic is to 1, the stronger the partial correlation between the variables and the better the factor analysis is. The KMO value for this part of the questionnaire was 0.735, as detailed in Table 8, and this value indicates suitability for factor analysis.

**Table 8:** Table of empirical scales Bartlett sphericity test and KMO test.

Number of KMO sampling suitability		
KMO and Bartlett tests		0.735
Bartlett’s spherical test	Cardinality last read DOF Significance	1357.370 45 0.000

Factor analysis was performed, and the extraction method of principal component analysis was used. Three common factors were extracted using an extraction method with eigenvalues greater than 1. The cumulative explained variance was 73.811%, which met more than 60% of the explanation criteria, and the results are shown in Table 9.

**Table 9:** Factor analysis of empirical scales.

Components	Total	Initial Eigenvalue Variance %	Total %	Total	Extraction of loading squares and variances	Total %	Total	Rotational load squared and variance	Total %
1	3. 175	31.751	31.751	3. 175	31.751	31.751	2.805	28.053	28.053
2	2.202	22.019	53.770	2.202	22.019	53.770	2.315	23. 151	51.204
3	2.004	20.041	73.811	2.004	20.041	73.811	2.261	22.607	73.811
4	0.557	5.574	79.384						
5	0.491	4.910	84.295						
6	0.397	3.973	88.267						
7	0.339	3.389	91.657						
8	0.322	3.216	94.873						
9	0.281	2.810	97.683						
10	0.232	2.317	100.000						

Extraction method: Principal component analysis.

To further clarify the structure of each common factor, the maximum variance method was used to orthogonally rotate the indicators, as detailed in Table 10. Let the first common factor be the functional experience factor, the second common factor be the emotional experience factor, and the third common factor be the social experience factor. From the factor loadings of the 10 measurement questions included in the three extracted factors, they were all above 0.5, which also exceeded the recommended critical level, reflecting the high structural validity of the questionnaire experience scale.

**Table 10:** Calculated results of orthogonal rotation of empirical scale indicators.

Rotating component matrix			
Functionality Experience 1	Component 1	Component 2	Component 3
Functionality Experience 2	0.825	0. 101	−0.005
Functionality Experience 3	0.813	0.070	0.058
Functionality Experience 4	0.852	0.073	0.064
Functionality Experience 1	0.838	0.041	0.009
Functionality Experience 2	0. 151	0.858	0.030
Functionality Experience 3	0.039	0.882	0.043
Social Experience 1	0.060	0.879	0.049
Social Experience 2	−0.005	0.039	0.841
Social Experience 3	0.075	0.067	0.883
	0.038	0.014	0.873

Extraction method: principal component analysis.

Rotation method: Kaiser standardized maximum variance method.

^a^Rotation converges after four iterations.

## 6. Results

**Figure 1:**
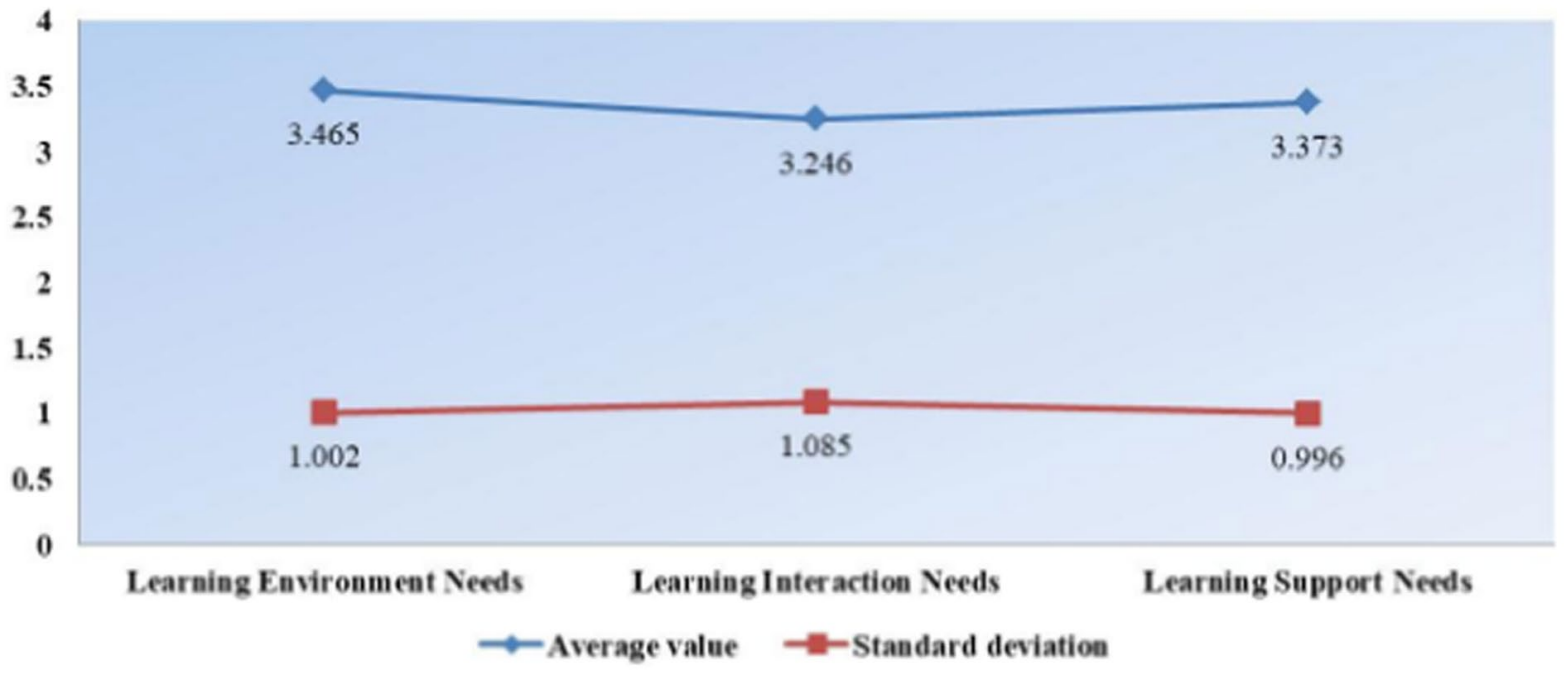
Statistical linear plot of learning needs.

As shown in Figure 1, according to the online learning demand statistics, it can be seen that the online network platform environment has the highest demand with a mean value of 3.465, followed by the learning platform support demand with a mean value of 3.373 and the lowest learning interaction demand with a mean value of 3.246. This indicates that students pay more attention to the learning environment of online learning courses, in other words, the learning environment helps students to learn better. In contrast, students’ need for learning interaction is not very high, which indicates that students pay less attention to interaction when using online sports learning courses on the platform, and the actual learning behavior is mainly based on learning knowledge itself.

**Figure 2:**
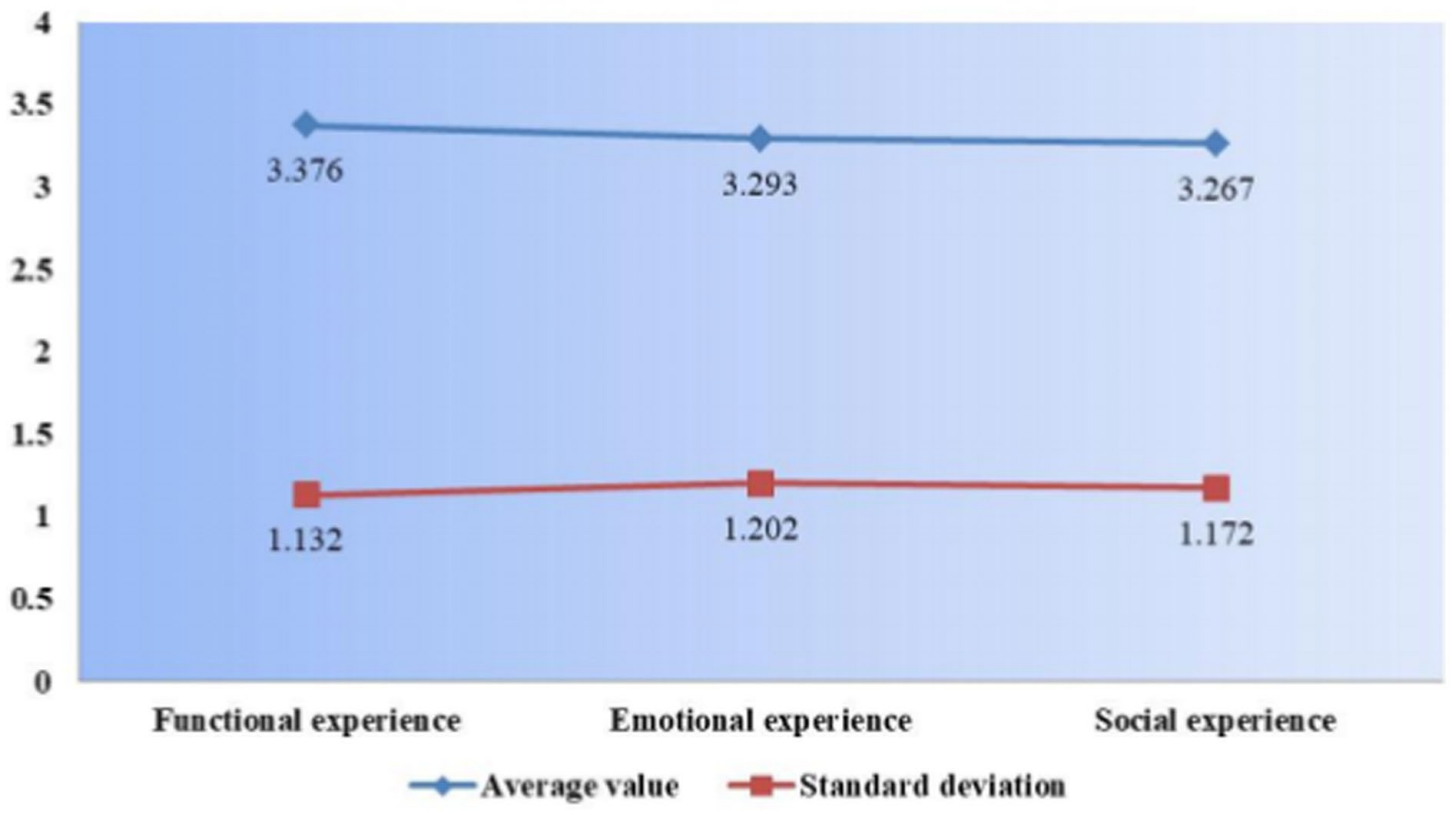
Statistical linear plot of learning experiences.

As shown in Figure 2, according to the statistical results of online learning experience, the online network platform has the highest functional experience with a mean value of 3.376, indicating that the online network education platform widely used by students now has better functions; the mean values of emotional experience and social experience are 3.293 and 3.267, respectively, indicating that the online network emotional experience and social experience are better, but both are lower than the online network platform functional experience.

**Figure 3:**
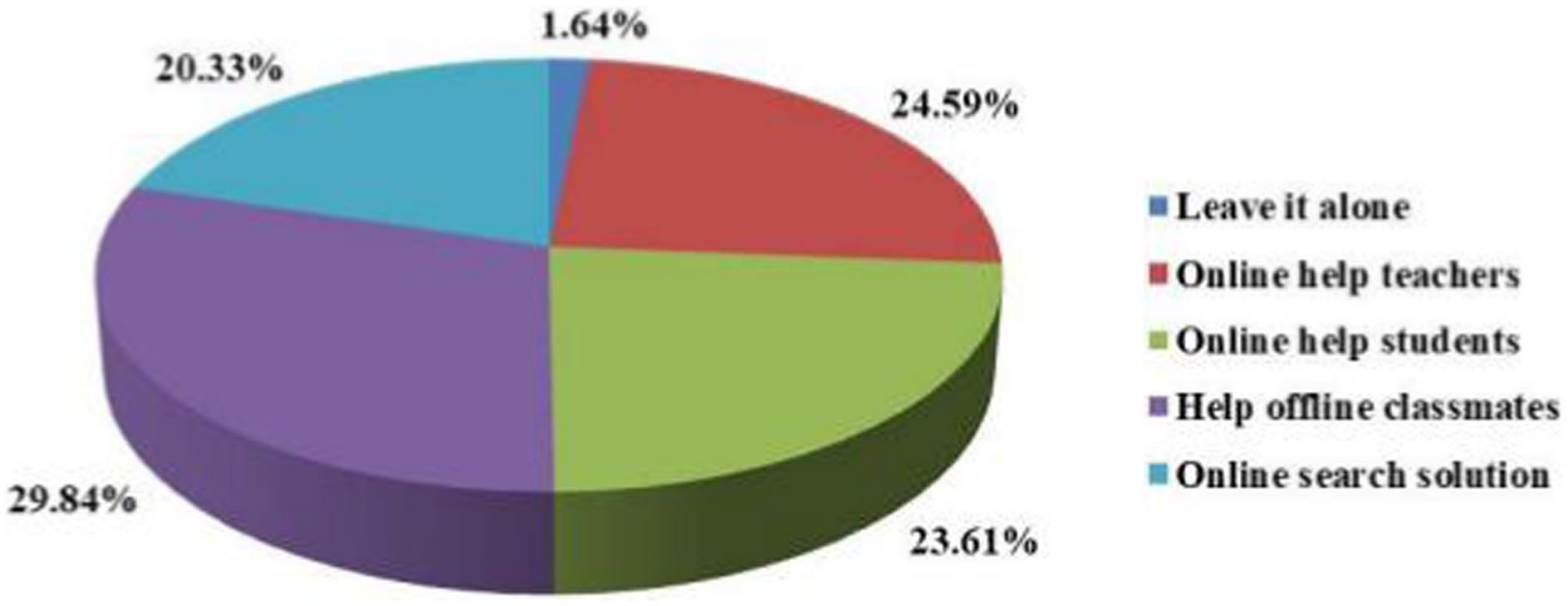
Solution statistics pie chart.

The results in Figure 3 show that 91 students used the “help offline classmates” method to seek help if they had a problem in an online course. There was little difference between the number of online instructors and online teachers, 75 and 72, respectively, and this was the second lowest solution for online search and solutions, with only 62 people choosing this option. A total of 209 people, or 68.52%, chose the online solution.

**Figure 4:**
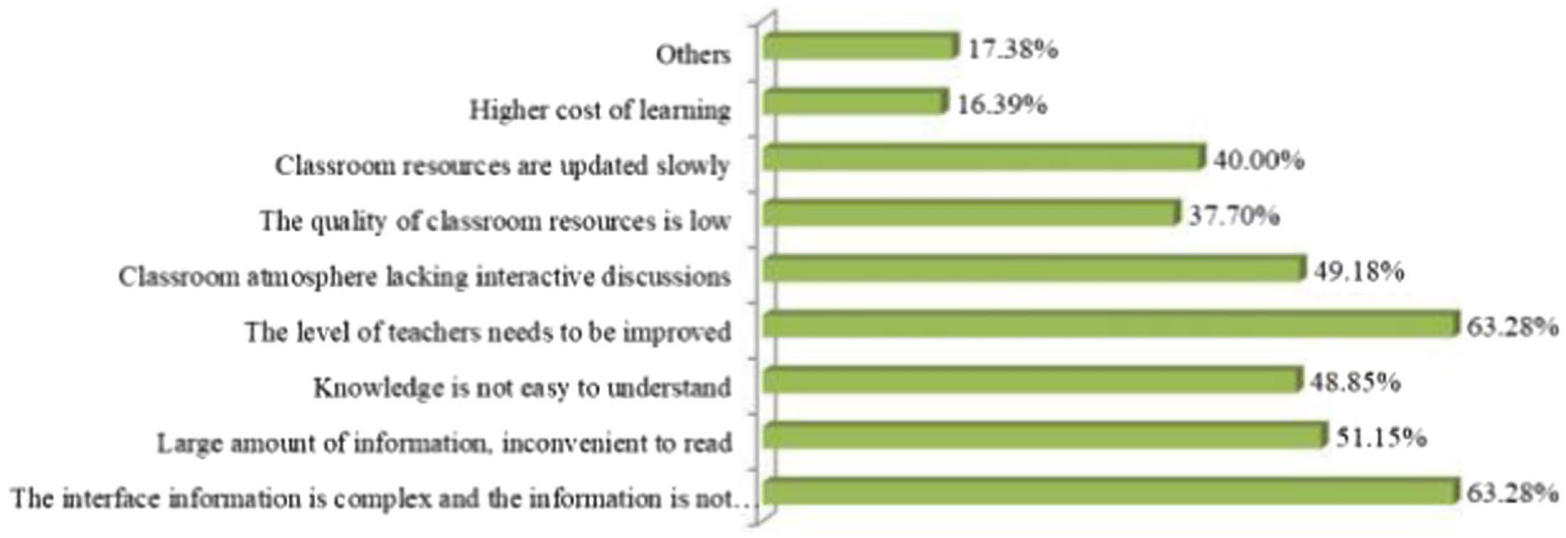
Statistical chart of problems with the online platform.

The analysis was performed using the chi-square goodness-of-fit test according to whether the proportion of each program was evenly distributed. As seen in Figure 4, the goodness-of-fit test showed significance (Chi = 167.499, *p* = 0.000 < 0.05), which means that the selection rate of each item was significantly different, and the differences can be specifically compared by response rate or penetration rate. Specifically, response rates and penetration rates were significantly higher for five items in the classroom atmosphere, including complex interface content, inconspicuous key information, large amount of information, inconvenient access, difficult knowledge to understand, teacher level to be improved, and lack of interactive discussion.

## 7. Conclusion

When people combine Internet information technology to break through the time and space of learning, the era of lifelong learning has quietly arrived. At present, the country vigorously promotes “Internet + Education,” digitalization and informatization of education. Only by keeping up with the update of information technology and constantly optimizing and providing high-quality network education resources can the physical education courses in colleges and universities avoid being outdated, decadent and abandoned by the times, and truly play their proper value, so as to improve the effect of physical education teaching.

The key to the integrated development of Internet online education platform and college sports is, firstly, the construction and upgrading of college sports online education resources; secondly, both platform users and auxiliary learners must have “Internet thinking” and use modern network technology to realize the efficient use of platform education resources; thirdly, it is to build a complete legal system to protect the rights and interests of sports online teaching resources from the legal level; and finally, it is clear that the closed loop of business interests to ensure sustainable development. However, from the actual situation in Chongqing, there are certain challenges in the construction of digital and informatized educational resources of college sports with strong practicality, and the informatization ability and professional strength of teachers responsible for the development and teaching of network resources still need to be improved; the corresponding platform also needs to be optimized in terms of resource information processing, interactive module design, and update timeliness. The independent learning and practice of physical education does not play a good supporting role, and the effect of online teaching of physical education is generally not optimistic.

Therefore, the construction and optimization of online sports resources to meet the needs of contemporary college students’ sports learning, and the development of hybrid teaching and personalized independent learning based on MOOC which meets the learning habits of contemporary learners, are essential to promote the development of modern online technology learning. Moreover, it is important for the development of university sports and the discipline itself to realize the reform of teaching mode and teaching reform.

## 8. Limitation

Due to the impact of the epidemic, this study only uses online questionnaire surveys, and the survey objects are college students majoring in physical education in Chongqing universities. Although the sample size is small, the schools and students covered are representative and can reflect the online education situation of Chinese college students, especially students majoring in physical education, under the epidemic.

## 9. Implications

According to the results of the questionnaire, this paper proposes the necessity of constructing and optimizing the online education resources of sports in colleges and universities. It is necessary to adapt to the characteristics of the “Internet+” era with advanced teaching rules. Keeping up with the pace of the times is the value concept. Innovate the traditional classroom teaching mode with relatively low efficiency to achieve the purpose of optimizing teaching effect and cultivating students’ independent learning, thinking and innovation ability. The characteristics of platform online education are proposed. The mainstream platform guarantees the user experience and the quality of teaching resources, online learning meets the needs of “Internet natives,” and the Internet provides possibilities for various learning conditions and environments.

## Data availability statement

The original contributions presented in the study are included in the article/supplementary material, further inquiries can be directed to the corresponding author.

## Author contributions

The author confirms being the sole contributor of this work and has approved it for publication.

## Funding

This work was funded by the Research Project of Counselor Work of Xi‘an Shiyou University “Study on ADR of student disputes in Chinese public universities from the perspective of improving the national security governance system and policy” (No. FYZ202101); and the Research Project of “Establishing Virtue and Cultivating People” of Xi‘an Shiyou University “Exploration and Practice of the Training Mode of Science and Engineering Compound Professionals Based on the Convergence Effect of Characteristic Platform Groups” (No. 140202103).

## Conflict of interest

The author declares that the research was conducted in the absence of any commercial or financial relationships that could be construed as a potential conflict of interest.

## Publisher’s note

All claims expressed in this article are solely those of the authors and do not necessarily represent those of their affiliated organizations, or those of the publisher, the editors and the reviewers. Any product that may be evaluated in this article, or claim that may be made by its manufacturer, is not guaranteed or endorsed by the publisher.

## References

[ref1] Asli M. F. Hamzah M. Ibrahim A. A. A. Ayub E. (2020). Problem characterization for visual analytics in MOOC learner's support monitoring: a case of Malaysian MOOC. Heliyon 6:e05733. doi: 10.1016/j.heliyon.2020.e05733, PMID: 33426320 PMC7775862

[ref2] Beard J. Konukman F. (2020). Teaching online physical education: the art of connection in the digital classroom. J. Phys. Educ. Recreat. Dance 91, 49–51. doi: 10.1080/07303084.2020.1785772

[ref3] Chen T. Peng L. Jing B. Wu C. Yang J. Cong G. (2020). The impact of the COVID-19 pandemic on user experience with online education platforms in China. Sustain. For. 12:7329. doi: 10.3390/su12187329

[ref4] Cruickshank V. J. Pill S. Mainsbridge C. (2021). 'Just do some physical activity': exploring experiences of teaching physical education online during Covid-19. Issues Educ. Res. 31, 76–93.

[ref5] Daum D. N. Goad T. Mosier B. Killian C. M. (2022). Toward quality online physical education: research questions and future directions. Int. J. Kinesiol. High. Educ. 6, 199–211. doi: 10.1080/24711616.2021.1930295

[ref6] De Notaris D. Canazza S. Mariconda C. Paulon C. (2021). How to play a MOOC: practices and simulation. Entertain. Comput. 37:100395. doi: 10.1016/j.entcom.2020.100395

[ref7] Erfayliana Y. Demirci N. Demirci P. T. (2022). Developing online modules for educators in fifth grade physical education class. JUMORA 2, 23–37. doi: 10.53863/mor.v2i1.420

[ref8] Goad T. Towner B. Jones E. Bulger S. (2019). Instructional tools for online physical education: using mobile technologies to enhance learning. J. Phys. Educ. Recreat. Dance 90, 40–47. doi: 10.1080/07303084.2019.1614118

[ref9] Hsu L. (2021). Learning tourism and hospitality subjects with massive open online courses (MOOCs): a cross-sectional and longitudinal study. J. Hosp. Leis. Sport Tour. Educ. 29:100276. doi: 10.1016/j.jhlste.2020.100276

[ref10] Lemes V. B. Fochesatto C. F. Brand C. Gaya A. C. A. Cristi-Montero C. Gaya A. R. (2022). Changes in children’s self-perceived physical fitness: results from a physical education internet-based intervention in COVID-19 school lockdown. Sport Sci. Health 18, 1273–1281. doi: 10.1007/s11332-022-00897-1 35126733 PMC8801194

[ref11] Li J. (2021). Application of mobile information system based on internet in college physical education classroom teaching. Mob. Inf. Syst. 2021, 1–10. doi: 10.1155/2021/1481070

[ref12] Ruiz-Palmero J. Fernández-Lacorte J. M. Sánchez-Rivas E. Colomo-Magaña E. (2020). The implementation of small private online courses (SPOC) as a new approach to education. International journal of educational technology. High. Educ. 17, 1–12. doi: 10.1186/s41239-020-00206-1

[ref13] Sosa-Díaz M. J. Fernández-Sánchez M. R. (2020). Massive open online courses (mooc) within the framework of international developmental cooperation as a strategy to achieve sustainable development goals. Sustain. For. 12:10187. doi: 10.3390/su122310187

[ref14] Tabuenca B. Kalz M. Löhr A. (2018). MoocCast: evaluating mobile-screencast for online courses. Univ. Access Inf. Soc. 17, 745–753. doi: 10.1007/s10209-017-0528-x

[ref15] Wegerif R. (2019). “Towards a dialogic theory of education for the internet age” in The Routledge International Handbook of Research on Dialogic Education (England: Routledge), 14–26.

[ref16] Yu J. Jee Y. (2020). Analysis of online classes in physical education during the COVID-19 pandemic. Educ. Sci. 11:3. doi: 10.3390/educsci11010003

